# Central Nervous System Biodistribution and Pharmacokinetics of Radiolabeled Tofersen in Rodents, Nonhuman Primates, and Humans

**DOI:** 10.2967/jnumed.125.270731

**Published:** 2026-01

**Authors:** Brendon E. Cook, Donald G. McLaren, Jenna M. Sullivan, Georges El Fakhri, Daniel L. Yokell, Mason W. Freeman, Nicolas Currier, Michael E. Oestergaard, Howard Dobson, Jacob Hesterman, Nicolas Salem, Ivan Nestorov, Michael Monine, Laurent Martarello, Karleyton C. Evans, Stephanie Fradette, Toby A. Ferguson, Danielle Graham, Luca Passamonti

**Affiliations:** 1 Biogen, Cambridge, Massachusetts;; 2 Departments of Medicine and Radiology, Massachusetts General Hospital, and Harvard Medical School, Boston, Massachusetts;; 3 Ionis Pharmaceuticals, Carlsbad, California; and; 4 Perceptive Discovery, Needham, Massachusetts

**Keywords:** antisense oligonucleotide, ALS, CSF, Qalsody, tofersen

## Abstract

Antisense oligonucleotides (ASOs) are an important therapeutic modality across several therapeutic areas, offering currently available and potential future treatment options for patients. ASO pharmacokinetics, biodistribution, and regional brain uptake are not fully characterized, particularly in humans. Here, we report preclinical studies and the first-in-human imaging trial measuring the biodistribution of [^99m^Tc]Tc-MAG3-tofersen. The tracer was designed to be a proxy for tofersen (Qalsody; Biogen), an ASO approved for the treatment of amyotrophic lateral sclerosis in adults who have a variant in the *SOD1* gene (*SOD1*-ALS). **Methods:** Tofersen was conjugated to a MAG3 moiety, which chelates ^99m^Tc to yield [^99m^Tc]Tc-MAG3-tofersen. [^99m^Tc]Tc-MAG3-tofersen and unlabeled tofersen were intrathecally injected in rats, nonhuman primates (NHPs), and healthy human volunteers (*n* = 3) via lumbar puncture, followed by SPECT/CT imaging. Tofersen was coadministered at a therapeutic dose. The tracer [^99m^Tc]Tc-MAG3-tofersen was prepared with greater than 99% purity. **Results:** Findings in rats demonstrated that [^99m^Tc]Tc-MAG3-tofersen was a proxy measure of unlabeled tofersen, and dosimetry was calculated from NHP imaging data. In a clinical study, unlabeled tofersen coadministered with a microdose of [^99m^Tc]Tc-MAG3-tofersen (≤129.5 MBq [3.5 mCi]) was well-tolerated. Human dosimetry estimates were within safe radiation dose levels. Imaging showed consistent distribution of radiolabeled ASO throughout the spinal cord and brain across species, with clearance patterns diverging in humans. Although rats and NHPs demonstrated declining brain concentrations over the study duration, human brain uptake increased during the first 4 h after injection. Additionally, tracer clearance from the spine in rodents and NHPs plateaued after 6 h but continued to decrease in humans. Radiolabeled ASO clearance from the lumbar spine was observed across all species, with peripheral clearance mediated primarily through the liver and kidneys. Broad uptake of the ASO in the brain and spinal cord is consistent with the clinical effects of tofersen observed in individuals with the *SOD1*-ALS variation. **Conclusion:** In preclinical and human SPECT/CT studies, [^99m^Tc]Tc-MAG3-tofersen mirrored unlabeled drug distribution, showing broad spinal cord and brain uptake, with some differences in kinetics among species.

## Visual Abstract

**Figure:**
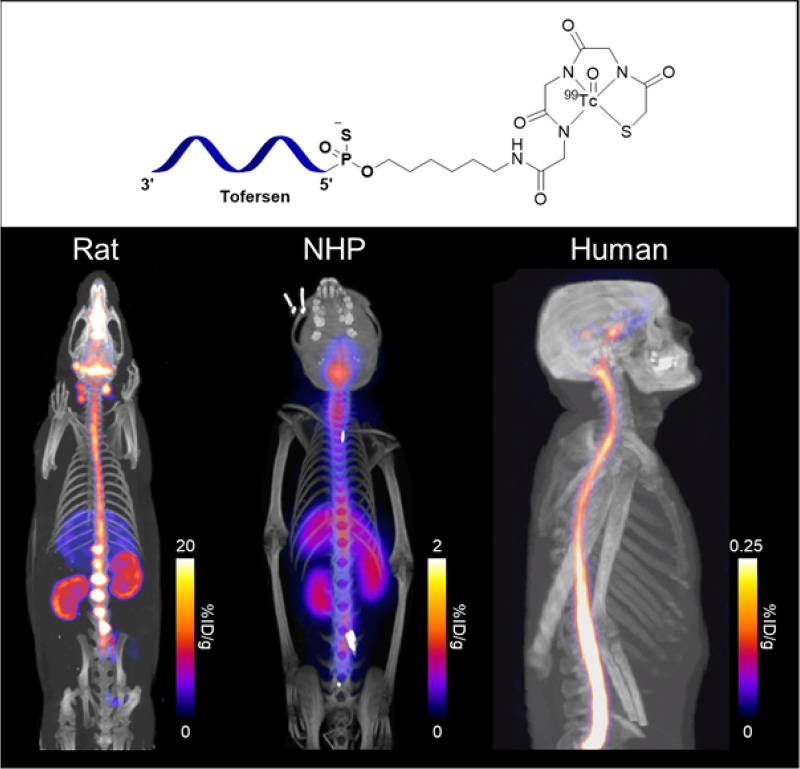

The antisense oligonucleotide (ASO) tofersen (Qalsody; Biogen) is the first approved therapy for the treatment of amyotrophic lateral sclerosis (ALS) in adults who have a mutation in the *SOD1* gene (*SOD1*-ALS). Tofersen-driven lowering of SOD1 protein has led to sustained reductions in neurofilament, a marker of neurodegeneration, and clinical benefit over time (1–7).

Tofersen biodistribution, and more generally ASO pharmacokinetics in the central nervous system (CNS), is not fully understood and has yet to be characterized in humans. For certain diseases and targets, the delivery of an ASO to key brain regions affected by disease pathology is anticipated to be necessary to elicit a robust therapeutic response. Thus, a greater understanding of ASO distribution and CNS pharmacokinetics can significantly inform the development of new ASOs targeting specific CNS areas, with selective vulnerability to different pathologies.

Previously, we developed a preliminary pharmacokinetic model of ASO distribution using nonhuman primate (NHP) data to predict ASO brain exposure in humans (8). This model predicts uptake in CNS tissues with concurrent clearance through the liver and kidneys. However, the challenges associated with validating this model through longitudinal in vivo assessments of ASO, particularly in humans, has hampered our understanding of ASO regional brain uptake and pharmacokinetics (9,10). The current study was designed to address this knowledge gap by conducting a series of radiochemical and preclinical experiments, as well as the first-in-human imaging trial to characterize in vivo tofersen biodistribution (Fig. 1).

**FIGURE 1.**
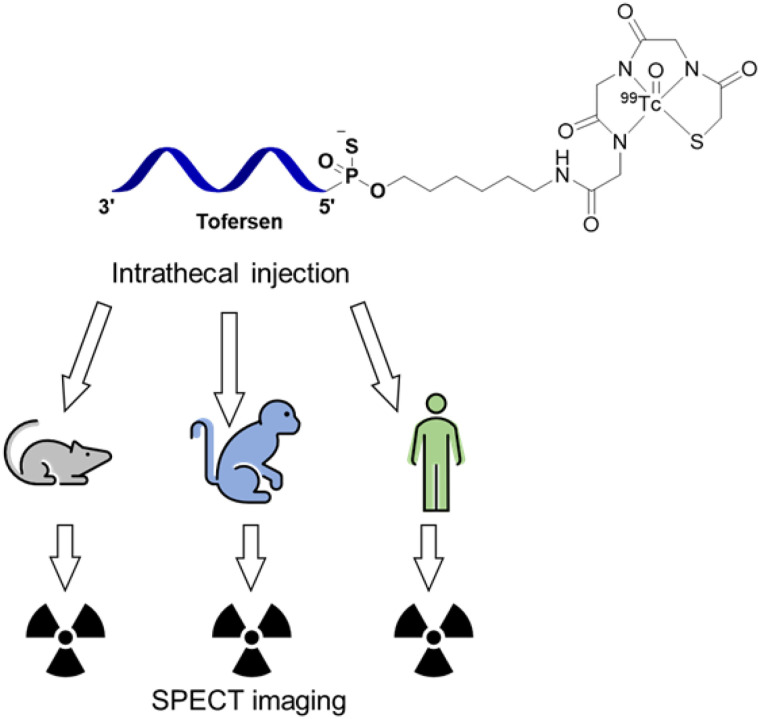
Tofersen was labeled with [^99m^Tc]Tc to enable SPECT imaging in rats, NHPs, and humans.

## MATERIALS AND METHODS

All animal studies were conducted in compliance with the U.S. Department of Agriculture’s Animal Welfare Act (9 CFR Parts 1–3) and followed the *Guide for the Care and Use of Laboratory Animal* (Institute of Laboratory Animal Resources). To ensure compliance, the study protocols were reviewed and approved by the respective Institutional Animal Care and Use Committee before animal receipt.

### Radiosynthesis of [^99m^Tc]Tc-MAG3-Tofersen

To facilitate radiolabeling with the SPECT isotope ^99m^Tc, the ASO tofersen was conjugated on its 5′ end with a MAG3 functional group, which is well-characterized for chelating technetium (Supplemental Fig. 1) (11,12). A short hexylamino spacer was used between the ASO and chelator to prevent steric interactions between the 2 components.

Bz-MAG3-tofersen (1 mg/mL, 100 μg) in phosphate buffer (100 μL, 0.5 M, pH 8.3) was mixed with citrate-phosphate buffer (100 μL, 0.1 M, pH 7.2), sodium tartrate solution (300 μL, 0.21 M), and a solution of ^99m^Tc-pertechnetate in saline (252 MBq [6.8 mCi], 200 μL). A freshly prepared aqueous solution of SnCl_2_ (5 μg in 50 μL of degassed saline) was immediately added, and the reaction was heated at 90 °C for 10 min and then allowed to stand at room temperature for an additional 30 min (Supplemental Fig. 1; available at http://jnm.snmjournals.org). The reaction was diluted with artificial CSF (aCSF), loaded onto an Amicon spin filter tube (3-kDa molecular weight cutoff), and buffer exchanged into aCSF. Radiolabeling resulted in 99% radiochemical purity. A portion of the purified product was concentrated to the required volume and further formulated with unlabeled tofersen, as needed.

### SPECT/CT Imaging in Rats

Treatment-naïve male Sprague–Dawley rats (*n* = 2) weighing 261 ± 3 g were anesthetized with isoflurane and surgically implanted with intrathecal catheters in the lumbar spine. The SPECT tracer [^99m^Tc]Tc-MAG3-tofersen (19.6 ± 0.1 MBq, 3 µg) and 240 µg of unlabeled tofersen were formulated in 31 µL of aCSF and injected via intrathecal catheter, followed immediately by a 40-µL saline flush. Each animal was scanned individually using whole-body SPECT/CT (Mediso Nanoscan) under isoflurane anesthesia in the prone position. Static SPECT images were acquired at 30 min and 1, 6, and 24 h after dose administration, followed immediately by a CT scan for anatomic reference.

### SPECT Imaging in NHPs

Two male and 2 female cynomolgus monkeys (*Macaca fascicularis*; 2.78 ± 0.36 kg) were included in the study. [^99m^Tc]Tc-MAG3-tofersen was prepared at high purity (>99%) and specific activity (3,700 ± 1,000 MBq/mg) at the end of synthesis and mixed with unlabeled tofersen (12 mg/dose) in aCSF (1.8 mL/dose).

Each animal was anesthetized with ketamine, maintained using isoflurane, and placed in the prone position. The area over the lumbar region was clipped and prepared with chlorhexidine scrub and solution. A 22-gauge spinal needle was introduced into the L3/L4 intrathecal space using aseptic technique. The placement of the needle was verified by the presence of cerebrospinal fluid (CSF) before and after dose administration. If required, the injection was administered at the intervertebral space rostral to L3/L4. Once placement of the needle was verified, the test article was slowly injected (37 ± 1.4 MBq in 1.8 mL of aCSF), and the needle was withdrawn. A predose and postdose count of the syringe, or dosing apparatus, was documented using the dose calibrator. A wipe of the dose site was performed and added to the postdose syringe count.

### Clinical Imaging Study in Healthy Volunteers

This was a phase 1, open-label, single-dose, imaging trial evaluating the safety, biodistribution, and regional brain uptake of a microdose (100 µg) of [^99m^Tc]Tc-MAG3-tofersen combined with unlabeled tofersen in healthy adult participants (Supplemental Table 5). The study was approved by the Massachusetts General Hospital Institutional Review Board and registered at ClinicalTrials.gov as NCT03764488. Written informed consent was obtained from each participant.

The study endpoints were the assessment of tofersen concentrations throughout the CNS by SPECT/CT imaging of [^99m^Tc]Tc-MAG3-tofersen and evaluation of the occurrence of adverse events (AEs) and serious AEs, as well as quantification of the radiation absorbed dose (dosimetry) in specific regions of the CNS.

The initial plan was to enroll approximately 8 healthy participants, with eligible participants to receive a single pharmacologically active dose of tofersen (up to 100 mg) containing up to 100 μg (no more than 129.5 MBq [3.5 mCi]) of [^99m^Tc]Tc-MAG3-tofersen in aCSF administered via intrathecal injection. The dose was administered in 15 mL of aCSF. Predose laboratory assessments were performed within 28 h before administration and reviewed before administration on day 1. To limit variability, and for consistency with ongoing and future studies of tofersen, a CSF sample of at least 10 mL was drawn from each participant before study treatment administration.

Two sites in the United States participated in this study; however, evaluable and nonevaluable data were collected, limiting the final scope of evaluable data to 3 subjects from a single site. Only a single study arm was conducted. Primary and secondary objectives were met despite the data collection limitations.

After a screening period of up to 35 d (including check-in), eligible participants checked into the site on day −1 (i.e., the day before the first treatment day) or day 1 (i.e., first treatment day) and were discharged after completing all assessments on day 2. Participants returned for follow-up visits on days 15 and 29. A 10-mL CSF sample was drawn from all participants on day 15 (postdose), and blood samples for determining immunogenicity were collected at check-in and on day 29. Participants received follow-up telephone calls on day 3 or 4 and days 8, 16, and 91. Unscheduled visits were conducted as needed for safety-related issues, at the investigator’s discretion.

## RESULTS

### Preclinical SPECT Imaging of [^99m^Tc]Tc-MAG3-Tofersen in Rats

SPECT imaging was performed in Sprague–Dawley rats with [^99m^Tc]Tc-MAG3-tofersen to evaluate its systemic and CNS distribution after intrathecal injection. [^99m^Tc]Tc-MAG3-tofersen (∼3 µg in mass) was coadministered with 240 µg of unlabeled tofersen and injected intrathecally in rats. This coadministration was performed in all in vivo experiments to enable pharmacokinetics measurements of the tracer, which would have been otherwise limited by active uptake via endocytosis and nonspecific binding of the tracer to CSF proteins (“carrier-added phenomena”) (13).

**FIGURE 2.**
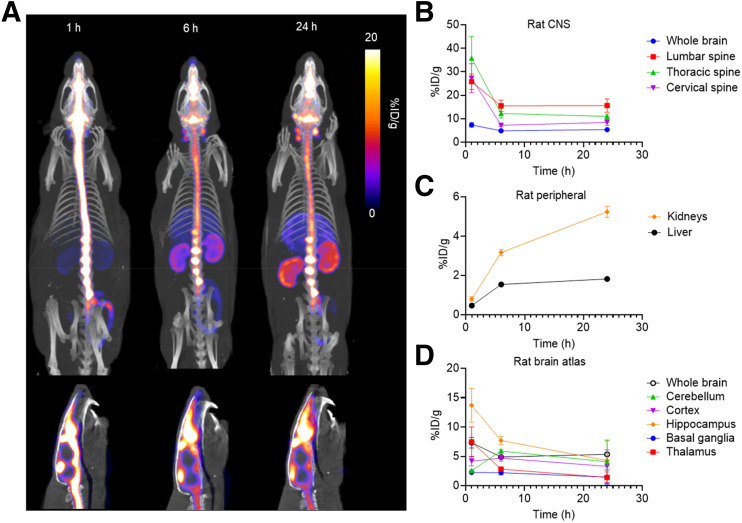
SPECT imaging of [^99m^Tc]Tc-MAG3-tofersen in rats. (A) Maximum-intensity-projection images of whole body (top) and sagittal views of head and cervical spine (bottom) showing distribution of [^99m^Tc]Tc-MAG3-tofersen in rats (*n* = 2) 1, 6, and 24 h after intrathecal injection. Time–activity curves derived from SPECT data measuring activity in ROIs in CNS (lumbar, thoracic, and cervical spine ROIs include both cord and proximal CSF) (B), kidneys and liver (C), and brain atlas (D).

After administration of the intrathecal dose, rats were imaged using SPECT/CT at 1, 6, and 24 h after injection. SPECT images demonstrated distribution along the spine and into the cranium, with peripheral uptake and clearance primarily in the liver and kidneys (Fig. 2A). Time–activity curves showed an initial clearance from the brain and spine within the first 6 h, with concentrations remaining mostly constant for 24 h (Figs. 2B–2D). Most brain subregions demonstrated clearance over the entire 24 h; however, an initial increase in uptake in the cerebellum was observed 1–6 h after injection, followed by a decrease in concentration between 6 and 24 h. Because of the SPECT scanner resolution, “spine” regions of interest (ROIs) reflect the combined signal from both the CSF and spinal cord.

### Validation of the Tracer by Ex Vivo Analysis

Observation of [^99m^Tc]Tc-MAG3-tofersen distribution is reliant on the integrity of the ^99m^Tc-MAG3 handle. Therefore, the tracer’s stability was tested in vivo. [^99m^Tc]Tc-MAG3-tofersen remained more than 95% intact in all tissues tested at 24 h postdose, with the exception of CSF (Supplemental Fig. 2). CSF sampling in rats showed greater variability at 1 h, primarily attributable to a single outlier. Detection in CSF and blood at 24 h was not possible because activity levels were below the detection threshold. These data support the assumption that in vivo SPECT images are representative of tracer distribution and that signal contribution from radiolabeled metabolites, if any, is not significant.

Next, it was critical to demonstrate that [^99m^Tc]Tc-MAG3-tofersen and unlabeled tofersen had equivalent kinetics and distribution in tissue to accurately estimate tofersen distribution with SPECT imaging. A biodistribution study was completed that measured both the absolute concentration of ASO in tissue by liquid chromatography–mass spectrometry and qualitative distribution using immunohistochemistry. The comparison was made between unlabeled tofersen and Re-MAG3-tofersen, a nonradioactive congener of the ^99m^Tc compound. At all time points, tofersen and Re-MAG3-tofersen concentrations did not significantly differ within the CNS regions sampled (Supplemental Fig. 3). Immunohistochemistry also showed similar brain distribution between the 2 compounds (Supplemental Fig. 4). The results provided confidence that measurements of radiolabeled tofersen represented the distribution of unlabeled tofersen.

### Tracer Kinetics and Dosimetry in NHPs

**FIGURE 3.**
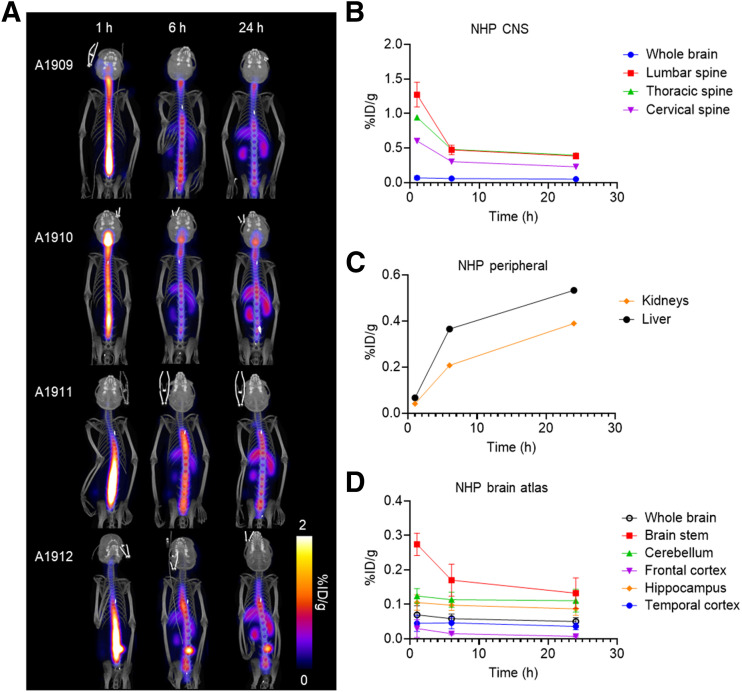
SPECT imaging of [^99m^Tc]Tc-MAG3-tofersen in NHP. (A) Maximum-intensity-projection images showing distribution of [^99m^Tc]Tc-MAG3-tofersen in NHPs (*n* = 4) 1, 6, and 24 h after intrathecal injection. Time–activity curves derived from SPECT data measuring activity in ROIs in CNS (lumbar, thoracic, and cervical spine ROIs include both cord and proximal CSF) (B), kidneys and liver (C), and brain atlas (D).

After successful testing in rats, an imaging study with [^99m^Tc]Tc-MAG3-tofersen and unlabeled tofersen was conducted in NHPs to evaluate the radiation dosimetry in preparation for testing in human participants. The intrathecally delivered tracer was initially distributed along the neuroaxis in the spine and brain, with peripheral activity increasing over time, primarily within the liver and kidneys (Fig. 3A). Among the 4 subjects, activity in the brain ranged from approximately 4% to 10% of the injected dose. Overall, clearance from the CNS was similar to that seen in the rats, although brain clearance was slower. The concentration of radioactivity was relatively consistent in the regions analyzed over 24 h, with the exception of the brainstem, which showed clearance over 24 h (Supplemental Fig. 5).

Dosimetry estimates were calculated using the SPECT imaging data. The OLINDA model estimated an effective mean ± SD dose of 4.98 ± 0.12 µSv/MBq, with the kidneys and liver having the highest absorbed dose of the organs tested (Supplemental Table 1) (14).

### Human Imaging Study in Healthy Volunteers

Healthy volunteers (*n* = 3) received an intrathecal dose of [^99m^Tc]Tc-MAG3-tofersen formulated with unlabeled tofersen in 15 mL of aCSF) after a 10-mL draw of CSF (consistent with the procedure for tofersen administration). Two participants received 103.4 or 109.5 MBq of [^99m^Tc]Tc-MAG3-tofersen with 100 mg of unlabeled tofersen. One participant (subject 1) received only 66.7 mg of tofersen in aCSF because of a formulation error (still in 15 mL of aCSF).

**FIGURE 4.**
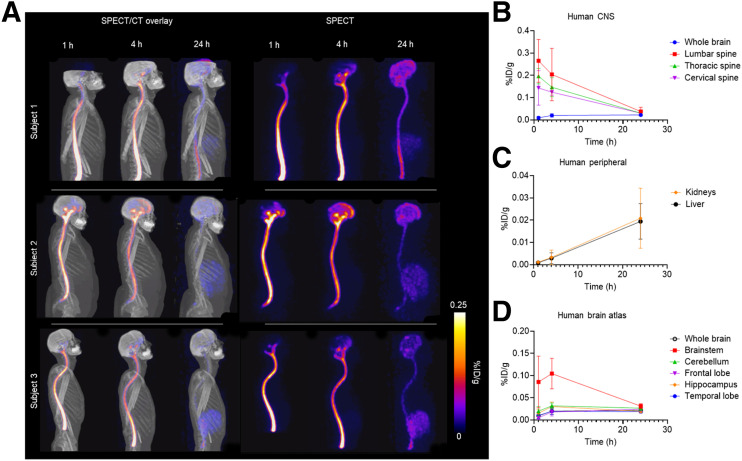
SPECT imaging of [^99m^Tc]Tc-MAG3-tofersen in healthy human volunteers. (A) Maximum-intensity-projection images showing distribution of [^99m^Tc]Tc-MAG3-tofersen in humans (*n* = 3) 1, 4, and 24 h after intrathecal injection. Time–activity curves derived from SPECT data measuring activity in ROIs in CNS (lumbar, thoracic, and cervical spine ROIs include both cord and proximal CSF) (B), kidneys and liver (C), and brain atlas (D).

Volunteers received whole-body SPECT/CT scans at 1, 4, and 24 h after injection. Maximum-intensity-projection images are presented as both SPECT/CT overlay or SPECT alone (Fig. 4A). Although the 4-h time point differed from the 6-h time point used in the NHP and rat studies because of logistic considerations, it was still deemed sufficient for calculation of dosimetry and tracer pharmacokinetics. At 24 h after injection, [^99m^Tc]Tc-MAG3-tofersen was distributed throughout the CNS in all participants and detected in all evaluated brain and spine regions. Corresponding animated maximum-intensity-projection images of each volunteer are provided in the supplemental materials.

Time–activity curves calculated from the CNS show clearance from all spine regions. However, unlike in the rat and NHP studies, clearance continued throughout the full 24 h of imaging rather than reaching equilibrium (Fig. 4B). Whole-brain uptake also differed, showing increasing activity between 1 and 4 h. This initial brain uptake can also be seen in the subregions analyzed (Fig. 4D).

The [^99m^Tc]Tc-MAG3-tofersen radioactivity concentration in the lumbar spine, lower thoracic spine, upper thoracic spine, and cervical spine peaked at 1 h after administration in 2 of 3 participants (subjects 2 and 3). The observed kinetics in the third participant (subject 1) differed in that the [^99m^Tc]Tc-MAG3-tofersen radioactivity peaked later in the upper thoracic and cervical spine (at 4 h after administration). In the atlas-based brain ROI [^99m^Tc]Tc-MAG3-tofersen radioactivity concentration peaked at 4 h after injection in 1 participant (subject 2) and peaked at 24 h after injection in the other 2 participants (subjects 1 and 3). [^99m^Tc]Tc-MAG3-tofersen radioactivity concentration in brain subregions varied over the 24-h period and across the 3 participants (Supplemental Fig. 6). In all participants, the atlas-based brain ROI [^99m^Tc]Tc-MAG3-tofersen radioactivity was lowest at 1 h after injection, and [^99m^Tc]Tc-MAG3-tofersen radioactivity in all spine regions was lowest at 24 h after injection.

Dosimetry was estimated for all participants (Supplemental Table 2). The effective dose—the weighted mean of the OLINDA/EXM 2.0 organ/tissue level absorbed doses weighted by each tissue’s sensitivity to radiation-induced damage—was 0.62 ± 0.112 mSv. None of the dosimetry estimates (for any organ or the whole body) exceeded radiation dose levels generally recognized as safe (21 CFR 361.1) (30 mSv for the whole body, active blood-forming organs, lens of the eye, and gonads and 50 mSv for single administration for other organs).

Whole-blood and serum samples for pharmacokinetics analysis were taken at 1, 2, 4, and 24 h after dose administration and summarized by the nominal pharmacokinetics sampling time, along with tofersen concentrations in serum as analyzed by bioanalytical assessment (Supplemental Table 3).

Whole-blood samples were analyzed via γ-counter for the concentration of [^99m^Tc]Tc-MAG3-tofersen, and serum samples were analyzed by enzyme-linked immunosorbent assay. For comparison with the radioactivity data, the serum pharmacokinetics data were normalized by the total injected dose (subjects 2 and 3) or 66.7 mg (subject 1) and converted to percentage injected dose per milliliter (Supplemental Table 3). The trajectory of individual profiles of whole-blood concentrations of tofersen as measured by radioactivity were consistent with the profiles of serum concentrations of tofersen. For the majority of time points, tofersen concentrations, as measured by radioactivity, were within 2-fold of corresponding serum concentration values.

Tofersen was generally well-tolerated when administered as 100 mg of unlabeled tofersen containing 100 µg of radiolabeled [^99m^Tc]Tc-MAG3-tofersen in 15 mL of aCSF. The most frequently reported AEs were procedural pain (reported by all 3 participants), followed by back pain (reported by 2 participants). Most AEs were mild in severity; 2 participants had AEs that were moderate in severity (1 participant with procedural pain and 1 participant with suicidal ideation and procedural pain). There were no serious AEs, and none of the AEs reported were considered related to the product under investigation, as assessed by the investigator. No AEs leading to treatment discontinuation or study withdrawal were reported. Furthermore, there were no treatment-emergent clinical laboratory abnormalities (including CSF parameters) or abnormal results in vital signs or 12-lead electrocardiograms that were reported as AEs.

The imaging data obtained were generally consistent across the 3 species studied. As the lumbar spine and lower thoracic spinal cord were closest to the site of injection, these regions had the highest concentration of radioactivity. Radioactivity remained comparatively lower in the brain, with initial delivery occurring before the 1-h time point in rats and NHPs. However, in human subjects, activity concentration in the brain increased over the first 4 h. Additionally, ASO continued to clear from the spine in humans over the 2-h study period, whereas the ASO plateaued after 6 h in rats and NHPs. The route of clearance was mostly consistent among the species, although NHPs displayed relatively higher uptake in the liver and kidneys.

## DISCUSSION

This study demonstrated that [^99m^Tc]Tc-MAG3-tofersen provides in vivo measurements of tofersen biodistribution and CNS pharmacokinetics after intrathecal administration. The study framework can be run with other ASOs, enabling direct measurements of drug distribution in otherwise inaccessible tissues and compartments to support drug development activities.

Initial experiments in rats verified that [^99m^Tc]Tc-MAG3-tofersen is a robust proxy measure for the biodistribution of therapeutic doses of tofersen, which was further validated in NHP and human studies. Evaluating the CNS and plasma distribution of both ^99m^Tc conjugated versus unconjugated tofersen, as well as the in vivo stability of the radiolabeled tracer, was critical for making this determination.

Across the 3 species tested, imaging distribution results with [^99m^Tc]Tc-MAG3-tofersen generally followed the same trend: after intrathecal injection, the tracer is well-distributed along the spine and into the brain, with heterogeneous distribution within different brain regions. This is followed by asymptotic clearance from the CNS, leading to uptake in the liver and kidneys.

However, when considering the tracer’s kinetics within the brain, the results in humans do not follow the same trend as that seen in rats and NHPs. In the human brain regions sampled, tracer concentration increased from 1 to 4 h before equilibrating, while in both rat and NHP brains, the signal decreased rapidly between 1 and 6 h. We hypothesize that this could be partly explained by the relative CSF volume differences among the 3 species and the volume of injection used for each. Although MRI measurements of CSF volume in rats and NHPs were not collected in this study, literature values report CSF volumes of approximately 0.6 mL in rats and approximately 12 mL in cynomolgus monkeys (15,16). Therefore, the total intrathecal injection volumes of 0.07 mL (rat) and 1.8 mL (NHP) accounted for approximately 11% and 15%, respectively, of their total CSF volume. In contrast, the mean ± SD CSF volumes measured by MRI in the healthy human participants was 410 ± 55 mL, whereas the injection volume of 15 mL after the removal of 10 mL of CSF (5-mL total volume added) accounts for approximately 1% of the total CSF volume (Supplemental Table 4). The impact of CSF withdrawal before dosing was not assessed and may contribute to observed differences. The nonclinical dosing volumes and protocols used in this study were based on published intrathecal injection standards shown to achieve robust rostral delivery of ASOs (16–18).

The data collected indicate that in humans, the injected dose requires more than 1 h to fully distribute throughout the brain, whereas in preclinical species, the larger relative injection volume results in more rapid dose distribution (16). This difference, if not properly accounted for in preclinical models, could result in an overestimation of the total injected dose that reaches the brain.

This study had several limitations. Despite its wide use, clinical SPECT has relatively low spatial resolution when compared with other molecular imaging techniques, such as PET. This limited our ability to assess and quantify ASO biodistribution and uptake in small brain regions such as the amygdala, brainstem, and specific parts of the spine (such as differentiating CSF from spinal cord and brain parenchyma). Resolution and partial-volume effects must also be considered when interpreting rat data due because of the small size of analyzed regions, such as the hippocampus.

Another shortcoming of this study was the relatively short half-life of the radioisotope used. Although this had clear advantages in terms of radiation exposure and logistic/operational aspects (particularly for the human study), the half-life of ^99m^Tc constrained our analysis to a 24-h window for quantification and assessment of ASO biodistribution. Tofersen and other ASOs have half-lives ranging from weeks to months; hence, future imaging experiments aimed at expanding our understanding of ASO pharmacokinetics will need to consider alternative imaging paradigms, such as longer-lived isotopes (e.g., ^89^Zr) or alternative imaging modalities, such as pretargeted imaging (19,20). The use of PET technology, particularly the new generation of scanners, can also increase the spatial resolution and signal quantification in small brain structures and minimize the radiotracer dose needed to acquire high-quality images (21,22).

## CONCLUSION

We demonstrated the feasibility and utility of radiolabeling for assessing in vivo biodistribution of [^99m^Tc]Tc-MAG3-tofersen. The broad uptake of the ASO in the brain and spinal cord in the healthy volunteer study is consistent with the clinical effects of tofersen observed in individuals with *SOD1*-ALS. Our data also confirmed that rodent and NHP models are suitable methods for preclinical testing of ASO-based tracers, enabling their deployment into human trials, although some differences in initial uptake and clearance from the CNS must be carefully considered. Collectively, these findings suggest that drug radiolabeling has potential as a technologic tool that can be used across a wide range of therapeutics to improve and refine our understanding of drug pharmacokinetics, biodistribution, and regional brain exposure.

## DISCLOSURE

All work was funded by Biogen. Brendon Cook, Donald McLaren, Jenna Sullivan, Nicolas Currier, Ivan Nestorov, Michael Monine, Laurent Martarello, Karleyton Evans, Stephanie Fradette, Toby Ferguson, Danielle Graham, and Luca Passamonti are or were Biogen employees and may hold stock in the company. Michael Oestergaard is an Ionis employee and may hold stock in the company. Jacob Hesterman was an employee of Invicro at the time this research was conducted. Mason Freeman was the principal investigator of the clinical study of tofersen in healthy human volunteers in the Translational and Clinical Research Centers at Massachusetts General Hospital (MGH) and received a sponsored research agreement award from Biogen, which was given to Massachusetts General Hospital. No other potential conflict of interest relevant to this article was reported.

## Notes

KEY POINTS **QUESTION:** Can [^99m^Tc]Tc radiolabeling enable noninvasive imaging of ASO biodistribution in the CNS? **PERTINENT FINDINGS:** In preclinical and human SPECT/CT studies, [^99m^Tc]Tc-MAG3-tofersen mirrored unlabeled drug distribution, showing broad spinal cord and brain uptake with some differences in kinetics among species. **IMPLICATIONS FOR PATIENT CARE:** This approach could guide and optimize ASO therapy development for neurodegenerative diseases, such as ALS.
